# Structural Repetition Detector: multi-scale quantitative mapping of molecular complexes through microscopy

**DOI:** 10.21203/rs.3.rs-5182329/v1

**Published:** 2024-10-15

**Authors:** Afonso Mendes, Bruno M. Saraiva, Guillaume Jacquemet, João I. Mamede, Christophe Leterrier, Ricardo Henriques

**Affiliations:** 1 Optical Cell Biology group, Instituto Gulbenkian de Ciência, Oeiras, Portugal; 2 Turku Bioimaging, University of Turku and Åbo Akademi University, Turku, Finland; 3 Faculty of Science and Engineering, Cell Biology, Åbo Akademi University, Turku, Finland; 4 InFLAMES Research Flagship Center, Åbo Akademi University, Turku, Finland; 5 Turku Bioscience Centre, University of Turku and Åbo Akademi University, 20520, Turku, Finland; 6 Department of Microbial Pathogens and Immunity, Rush University Medical Center, Chicago, Illinois, USA; 7 Aix Marseille Université, CNRS, INP UMR7051, NeuroCyto, Marseille, France; 8 UCL Laboratory for Molecular Cell Biology, University College London, London, United Kingdom; 9 Instituto de Tecnologia Química e Biológica António Xavier, Universidade Nova de Lisboa, Oeiras, Portugal

**Keywords:** structural biology, quantitative image analysis, super-resolution microscopy

## Abstract

From molecules to organelles, cells exhibit recurring structural motifs across multiple scales. Understanding these structures provides insights into their functional roles. While superresolution microscopy can visualise such patterns, manual detection in large datasets is challenging and biased. We present the Structural Repetition Detector (SReD), an unsupervised computational framework that identifies repetitive biological structures by exploiting local texture repetition. SReD formulates structure detection as a similarity-matching problem between local image regions. It detects recurring patterns without prior knowledge or constraints on the imaging modality. We demonstrate SReD’s capabilities on various fluorescence microscopy images. Quantitative analyses of three datasets highlight SReD’s utility: estimating the periodicity of spectrin rings in neurons, detecting HIV-1 viral assembly, and evaluating microtubule dynamics modulated by EB3. Our open-source ImageJ and Fiji plugin enables unbiased analysis of repetitive structures across imaging modalities in diverse biological contexts.

## Introduction

**Fig. 1.**
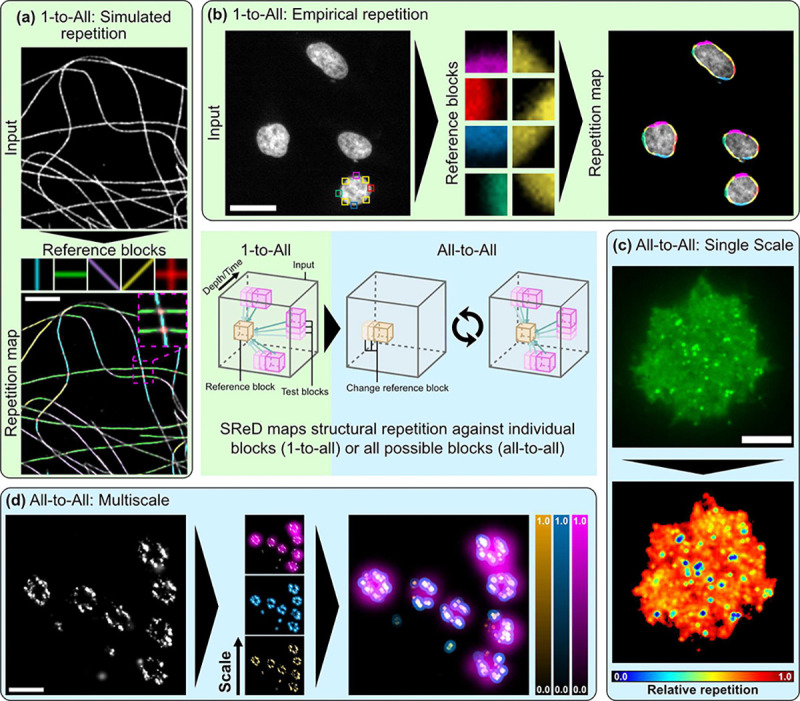
Applications of the Structural Repetition Detector (SReD) Algorithm in Fluorescence Microscopy. **a**, Detection of Structural Repetition Using Simulated Blocks: Microtubules imaged with STORM analysed for repetitive patterns using simulated structural blocks. Coloured regions in repetition map correspond to repetitions of same-coloured blocks above. **b**, Detection of Structural Repetition Using Empirical Blocks: HeLa cell nuclei stained with DAPI used to detect repetitive structural patterns using manually extracted empirical blocks. Coloured regions in repetition map correspond to repetitions of same-coloured blocks in previous subpanel. **c**, Global Repetition Detection: Jurkat cell expressing inducible HIV-1 Gag-EGFP fusion protein analysed using global repetition detection. Image probed for structural repetition using all possible empirical patches. Repetition map reveals structures not easily detectable in input image and their relative frequency. **d**, Multiscale Global Repetition: *Xenopus laevis* nuclear pores imaged with STORM analysed using different-sized receptive fields to detect structural repetition at various scales. Repetition map identifies repeated structures from single nucleoporins (orange) to nucleoporin clusters (blue) and nuclear pore units (magenta). Centre panel: Simplified SReD algorithm workflow, illustrating key steps from input preprocessing to repetition map generation.

Biological systems exhibit structural repetition across multiple scales, from biomolecules to supramolecular assemblies and cellular structures (1). Understanding these patterns is crucial for identifying their functional significance and underlying biological processes (2). Microscopy techniques offer molecular-level resolution but manually detecting repetitive motifs in large datasets is impractical, biased, and expertise-dependent (3). To address these limitations, machine learning, particularly deep convolutional neural networks (CNNs), has been employed to detect and segment biological structures automatically (4, 5). However, CNNs require extensive labelled training data, inheriting biases (6). Previous methods enable unbiased registration but need single-molecule localisation data, limiting their applicability (7, 8). We present the Structural Repetition Detector (SReD), an unsupervised framework to identify repetitive biological structures by exploring local texture redundancy. SReD formulates structure detection as similarity matching between local image regions, allowing pattern detection without prior knowledge or microscopy modality constraints. We demonstrate SReD’s capabilities on fluorescence microscopy images of diverse cell types and structures, including microtubule networks, nuclear envelope, pores, and virus particles (Fig. 1). SReD generates Structural Repetition Scores (SRSs) highlighting regions with repetitive textures. Users can provide artificial blocks or extract them from the data for repetition analysis. An unbiased sampling scheme maps global repetition by testing every possible image block as a reference (**Note S1**). We showcase SReD’s utility through three datasets: 1) spectrin rings in neuronal axons, accurately estimating ring periodicity and pinpointing periodic patterns, 2) HIV-1 Gag assembly, mapping viral structures without structural priors, and 3) dynamic EB3 and microtubule structures, assessing structural displacement and stability over time. Our open-source ImageJ and Fiji plugin enables versatile, unbiased analysis of redundancy in microscopy images. SReD advances computational microscopy by providing a generalised framework for detecting repetitive structures without labelled training data or single-molecule localisation input, facilitating the quantitative study of structural motifs across scales in diverse imaging datasets.

## Results

### General applications of SReD.

SReD is an open-source ImageJ and Fiji plugin that leverages GPU acceleration to identify repetitive patterns in microscopy images. The algorithm’s workflow, outlined in Fig. 1 (centre panel) and **Note S1**, begins with the application of the Generalised Anscombe Transform (GAT) to stabilise noise variance (9). This step addresses the noise in microscopy images, which often exhibit Poisson and Gaussian noise. The GAT nonlinearly remaps pixel values to produce an image with near-Gaussian noise and stabilised variance, preserving local contrast and overall image statistics. This stabilisation is essential for robust downstream processing, mitigating violations of normality, homoscedasticity, and outlier assumptions that can compromise correlation metrics. Following noise stabilisation, SReD generates a relevance mask to exclude regions lacking substantive structural information (**Note S1; Fig. S1**). The analysis proceeds using reference blocks, either simulated or empirically sampled from the image. These blocks are matched against the input using correlation metrics to generate repetition maps, as detailed in the Methods section. The resulting repetition map highlights regions likely to contain structural repetitions, with nonlinear mapping applied to visually emphasise salient features. To demonstrate SReD’s versatility across diverse biological contexts, we conducted a comprehensive analysis of various microscopy datasets (Fig. 1; **Note S2**). We first examined a STORM image reconstruction of a cell with labelled microtubules (10). This approach effectively mapped microtubules at various orientations and crossings (Fig. 1a; **Fig. S2**). We further illustrate SReD’s versatility by detecting nuclear envelopes in DAPI-stained cells (11) using empirical reference blocks extracted directly from the input image, distinguishing different morphological states potentially related to cell division or stress (Fig. 1b; **Fig. S3**). SReD also enables characterisation of structures without user-provided references. We exemplify this functionality by analysing an image of a Jurkat cell expressing an HIV-1 Gag-EGFP construct, which induces the production of virus-like particles (VLPs) (Fig. 1c; **Fig. S4**). In this mode, SReD mapped every structure in the image and assigned scores based on their relative repetition. As expected, the top score was given to the most repeated element, the diffuse eGFP signal, with viral structures exhibiting lower frequencies. Localisation of round viral structures via local extrema calculation revealed that the repetition map provided a superior platform for extrema detection compared to direct analysis of the raw images. The algorithm’s multiscale analysis capability is achieved by adjusting the ratio of block-to-image dimensions. Larger ratios capture larger structures, while smaller ratios capture finer details. For computational efficiency, it is preferable to modulate scale by downscaling the input rather than enlarging blocks, although combining both approaches often preserves structural detail best. We demonstrate this multiscale analysis by examining nuclear pore complexes in STORM image reconstructions with labelled gp210 proteins (12). SReD successfully mapped structures across different scales, discerning single nucleoporins, nucleoporin clusters, and entire nuclear pores (Fig. 1d; **Fig. S5**).

### 1-to-all case example: detection of spectrin ring periodicity in axons.

**Fig. 2.**
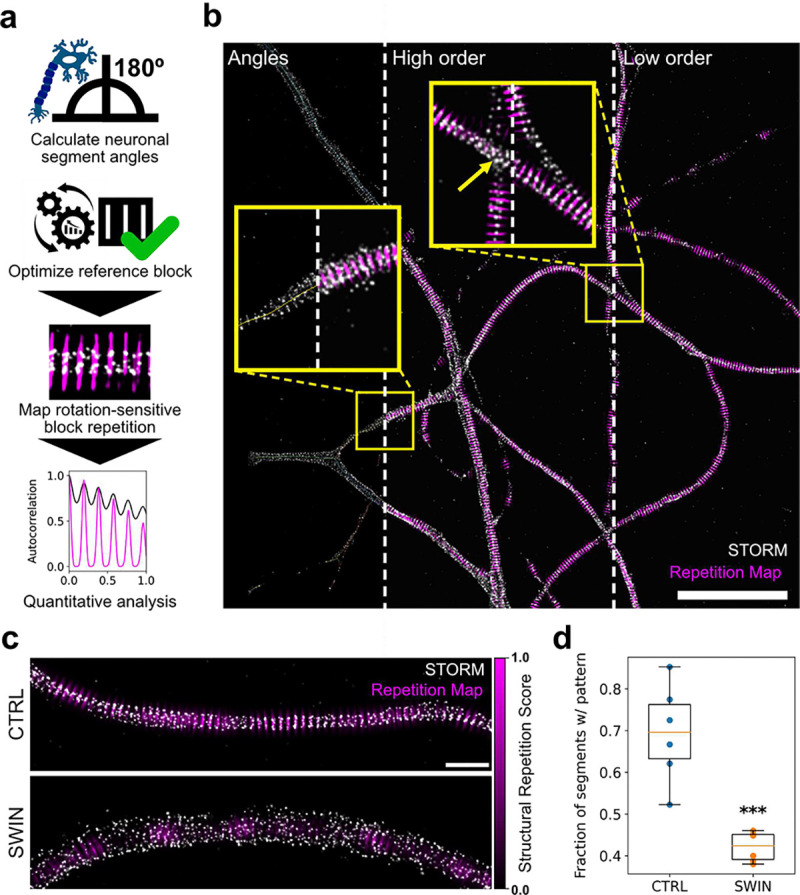
Automated detection and quantification of spectrin ring periodicity in neuronal axons. **a**, SReD-based analysis pipeline: the algorithm determines axon orientations, optimises a reference block for spectrin rings, and maps structural repetitions. Quantitative analysis is performed using autocorrelation and other methods. **b**, Control dataset image: STORM localization density (grey) overlaid with SReD repetition map (magenta). Insets: (i) ‘Angles’ - axon skeletons colour-coded by orientation; (ii) ‘High order’ - repetition map with a 9-ring reference block; (iii) ‘Low order’ - repetition map with a 3-ring reference block. Scale bar: 5 μm. **c**, Repetition maps comparing control (CTRL) and swinholide A-treated (SWIN) groups. SWIN-treated samples show reduced periodic structures. Scale bar: 1 μm. **d**, Quantification of axon segments with ring patterns. Box plot shows a significant reduction in pattern-containing segments in SWIN vs. CTRL (n=6 per group, mean±SEM; CTRL: 0.694±0.008, SWIN: 0.421±0.007; p<0.001, unpaired t-test).

We used SReD’s block repetition mode to map and quantify the membrane-associated periodic scaffold (MPS) architecture in neuronal axons automatically and without bias (Fig. 2). The MPS, composed of actin, spectrin, and associated proteins, forms a crucial structural component of neuronal axons (13, 14). Super-resolution microscopy has shown that the MPS consists of ring-like structures spaced 180–190 nm apart, with alternating actin/adducin and spectrin rings orthogonal to the axon’s long axis (15–18). Mapping this nanoscale organisation across entire neuron samples has been challenging due to the need for manual region selection, potentially introducing bias. We analysed datasets from Vassilopoulos *et al.* (16), comparing neurons treated with DMSO (control) or swinholide A (SWIN, an actin-disrupting drug). Using SReD, we developed an automated workflow to determine axon orientations by probing skeletonised neuron images with simulated lines at varying angles (**Note S3; Fig. S6**). This enabled consistent alignment of axon segments for downstream analysis. We optimised parameters for simulated ring blocks to match observed ring patterns in control data, yielding an inter-ring spacing of 192 nm, consistent with previous studies (**Fig. S7**)(15–17). SReD generated repetition maps highlighting regions of high local similarity across neuron samples, allowing automatic extraction and quantification of MPS organisation without manual region selection (Fig. 2b; **Fig. S8**). We measured an average spacing of 178 nm under control conditions (Fig. 2c; **Fig. S9d**). In agreement with Vassilopoulos et al. (16), repetition maps showed that swinholide A treatment disrupted MPS structure, with reduced pattern prominence and frequency compared to controls (Fig. 2b,c). We used correlation metrics that minimise information loss while being aware of potential imprinting. Nonlinear mapping effectively distinguishes real patterns from imprinted ones (**Fig. S2d,e; Fig. S9c**). Our method accounts for neuron thickness variability and provides the average distance between patterns for additional biological insights. SReD’s local repetition scores quantified the fraction of structures with MPS patterns, revealing a 39% reduction in axons with detectable periodic scaffolds after swinholide A treatment (P<0.001, Fig. 2d). SReD’s maps identified drug-affected regions with confidence values, offering a detailed platform for analysing structural dysregulation (Fig. 2c). SReD also showed higher statistical sensitivity, detecting a 12% reduction in pattern prominence post-treatment (P<0.05) previously unreported (**Fig. S9e**). To test SReD’s noise robustness, we conducted a sensitivity analysis with images at varying signal-to-noise ratios (SNRs) (**Fig. S10**). SReD consistently detected ring structures even at low SNRs near 1, where patterns were visually indiscernible. SReD-generated maps outperformed direct STORM reconstructions in autocorrelation analysis, reliably identifying an average inter-ring spacing of 192 nm across all SNRs, demonstrating the algorithm’s robustness in detecting structural periodicity despite significant noise. We assessed SReD’s specificity and robustness to pattern deformations by applying stretch deformations to test images (**Fig. S11**). As the stretch factor increased, the average SRS decreased, indicating pattern disruption. However, SReD remained specific to the original pattern within the expected interval. Even at higher stretch factors, non-specific patterns were quantitatively discernible and reflected the intrinsic properties of the test data. This robustness is valuable for analysing periodic structures in diverse biological contexts, where deviations from ideal patterns are common due to sample preparation artefacts, imaging noise, or biological variability.

### All-to-all 3D case example: detecting HIV-1 Gag assembly in 3D.

**Fig. 3.**
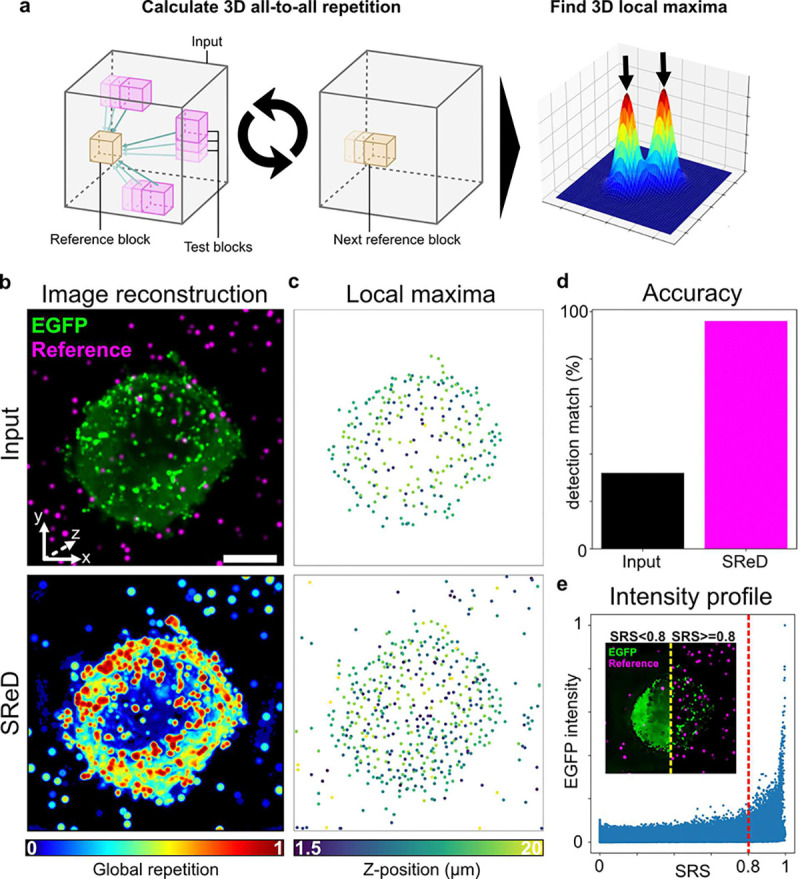
Detecting HIV-1 Virus-Like Particles in 3D. **a**, Analysis pipeline schematic. The algorithm uses 3D reference blocks for structural repetition analysis, locating viral structures via local maxima from the input image and repetition map. **b**, Z-projections of the input image (left) and repetition map (right), highlighting viral-like particles (“EGFP”) and simulated reference particles (“Reference”). **c**, Local maxima plots showing detected structures in the input image (left) and repetition map (right), with increased sensitivity in the repetition map. **d**, Accuracy plot comparing artificially added reference particle detection: input image (32%) vs. repetition map (96%). **e**, Intensity profile graph of EGFP signal (green) and structural repetition score (SRS, magenta), with a threshold at SRS 0.8 (dashed red line). Inset shows pixels below (dark) and above (light) the threshold, indicating high-SRS structures. Scale bars: 5 μm (main images), 1 μm (insets).

The establishment of a viral infection is the product of complex host-pathogen interactions, comprising an evolutionary “tug-of-war” where cells evolve protective mechanisms whilst viruses evolve to circumvent them. Viruses typically hijack cellular transcription and translation machinery to produce viral progeny required for viral replication (19). Therefore, viral assembly represents a critical platform for host-pathogen interactions that significantly impact infection outcomes. The HIV-1 *gag* gene encodes the Gag polypeptide precursor, which is cleaved into several key structural components. This polypeptide aggregates at the membrane of infected cells and induces the budding of membranous viral particles (19). Expression of Gag alone is sufficient to induce the formation of non-infectious virus-like particles (VLPs)(20, 21). To map viral structures in an unbiased manner, we examined an image of a Jurkat cell expressing an inducible HIV-1 Gag-EGFP construct using SReD (Fig. 3a,b). To evaluate the algorithm’s accuracy, we generated a population of simulated diffraction-limited particles with randomly distributed intensities across the image’s dynamic range, which served as a reference for comparison. Local maxima corresponding to active viral assembly sites were calculated from both the input image and the repetition map using identical parameters (Fig. 3c). Remarkably, SReD enabled the detection of 96% of the simulated particles, compared to only 32% in the input image, demonstrating the algorithm’s superior accuracy over direct analysis of input images (Fig. 3d). Visual inspection of the detected EGFP intensity signal vs. the SRS for the same pixel location revealed that high SRS regions corresponded to input regions with a wide range of intensity values. We observed that most structures of interest were allocated to the sample fraction above an arbitrary threshold of SRS 0.8, whilst the fraction below this threshold contained mostly background signal and some reference particles (Fig. 3e). Given that autofluorescence often corrupts microscopy analyses, we evaluated the algorithm’s performance in the presence of synthetic non-specific structures (**Note S4**). The repetition maps produced by SReD consistently provided a superior platform for detecting simulated reference particles and viral structures across conditions (**Fig. S12**). This analysis demonstrates SReD’s robust capability to map biological structures, such as assembling viral particles. The algorithm’s high sensitivity and specificity, even in the presence of non-specific structures, highlight its potential for studying dynamic cellular processes like viral assembly, where the ability to accurately detect and characterise structures amidst variable backgrounds is showcased.

### All-to-all live-cell case example: assessment of microtubule dynamics.

**Fig. 4.**
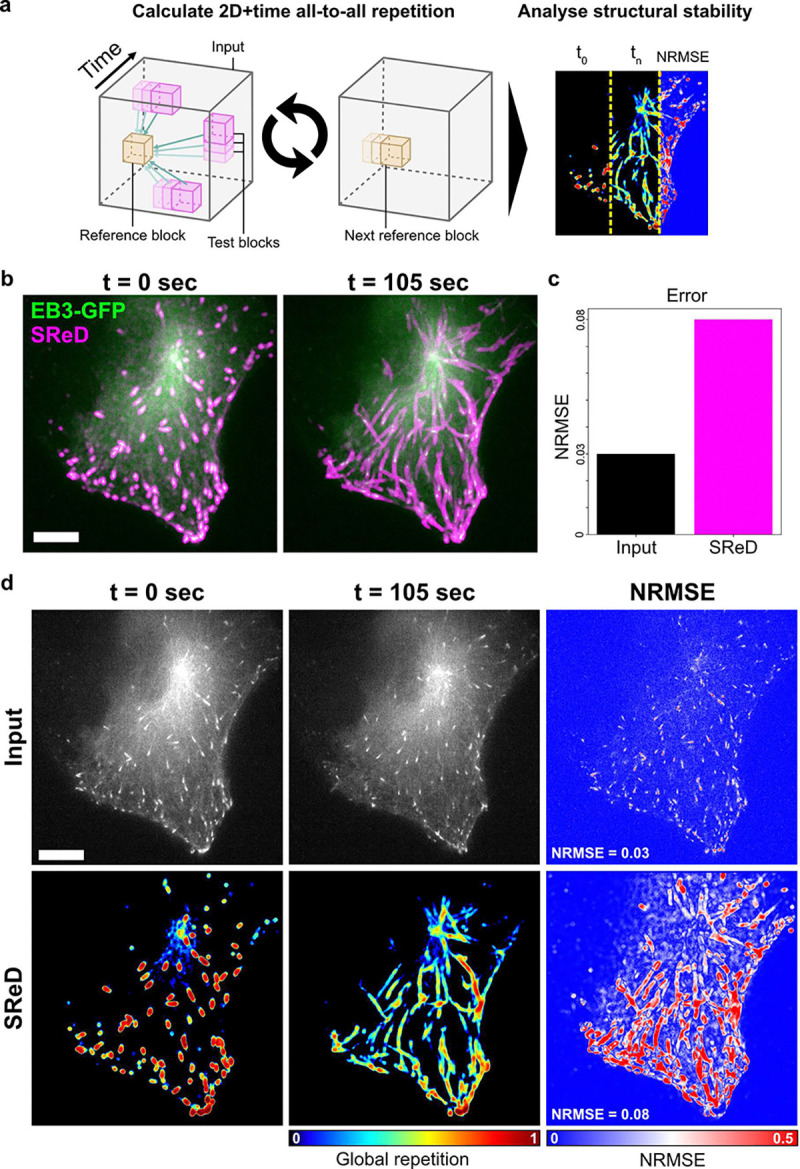
Assessment of microtubule dynamics using SReD. **a**, Analysis pipeline schematic. Global repetition analysis used time as the third dimension on a timelapse sequence of RPE1 cells expressing EB3-GFP over 105 seconds (35 frames). The first frame served as the control. Normalised Root Mean Squared Error (NRMSE) quantified structural differences between time points. **b**, Input images (left) and global repetition maps (right). The first frame’s repetition map highlights EB3 comets, while the entire time-lapse map shows comet trajectories and repetition over time. **c**, Bar graph of average NRMSE between input images and repetition maps. Higher error in repetition maps (0.08) vs. control images (0.03) indicates greater sensitivity to structural changes. **d**, NRMSE maps of input images (left) and repetition maps (right), showing structural stability over time. High NRMSE values (warmer colours) in EB3 trajectories indicate lower stability, while lower values (cooler colours) in the Microtubule Organising Centre (MTOC) indicate higher stability. Scale bars: 10 μm (main images), 2 μm (insets).

The multidimensional capabilities of SReD can be extended to analyse structural dynamics over time, providing insights into structural stability. We demonstrate this application using time-lapse imaging of RPE1 cells stably expressing End-binding Protein 3 (EB3) fused to GFP (Fig. 4a). EB3 binds to the plus ends of microtubules, appearing as comet-like structures that travel along the cytoplasm when visualised under fluorescence microscopy (22). We generated a global repetition map by treating time as the third dimension in our analysis, using a time-lapse sequence of approximately 2 minutes (Fig. 4b). To quantify structural changes, we calculated the Normalised Root Mean Squared Error (NRMSE) between the first and last frames of the time-lapse for both the input images and the repetition maps. The NRMSE of the input images reflected the spatial displacement of dynamic structures, yielding a relatively low value. In contrast, the NRMSE calculated from the repetition maps was substantially higher, indicating greater sensitivity to structural changes over time (Fig. 4c). SReD effectively mapped the spatial distribution of EB3 comet activity over time. By quantifying the repetitiveness of structures, it assigned scores to different regions, highlighting areas with high EB3 comet presence and their trajectories. The NRMSE maps further emphasised this distinction, revealing elevated values along comet paths, indicative of their dynamic nature. In contrast, the MTOC demonstrated notably lower NRMSE, suggesting its greater stability compared to the more mobile EB3 comets (Fig. 4d). The time interval used in the analysis captures the relatively slower dynamics of EB3 comets in this context. While individual comet tracking is not the primary focus of this method, the approach effectively reveals the spatiotemporal stability of structures, where instability often results from displacement, visually manifesting as comet trajectories. To further validate our approach, we performed the analysis with increased temporal resolution. We compared SReD’s results with conventional time projections of the input data, revealing advantages of our method. Unlike time projections, which typically integrate local intensities across time, SReD calculates local correlations of images across time, providing relative repetition scores that indicate how much the texture at each location changes relative to all other textures. This approach offers two significant benefits: (i) it provides a more nuanced measure of structural stability over time, and (ii) it is less susceptible to noise and intensity in-consistencies across time points (**Note S5; Fig. S13**). In this type of combined spatial and temporal analysis, instead of producing a time series, SReD’s output is a single map that shows the local stability of the timelapse over a specific time interval. This representation offers a comprehensive view of the structural dynamics that is not easily achieved using traditional methods such as kymographs. While kymographs are useful for tracking individual structures over time, SReD provides a broader perspective on the overall stability and dynamics of subcellular structures across the entire field of view.

## Discussion and Conclusions

Our results demonstrate SReD’s versatility and analytical power across diverse biological contexts. In neuronal axons, SReD enabled automated, unbiased mapping of the membrane-associated periodic scaffold (MPS), revealing nuanced changes in pattern frequency and prominence following pharmacological perturbation. While previous studies by Vassilopoulos et al. (16) reported a 40% reduction in overall MPS prominence after treatment with swinholide A, SReD’s analysis provides a more detailed characterisation of the phenotype. By first distinguishing between regions with and without periodic patterns, and then analysing only the pattern-present areas, SReD detected a 12% reduction in pattern prominence and a 32% reduction in pattern frequency. This refined analysis not only corroborates the previously reported overall effect but also decomposes it into two distinct components, offering deeper insights into the nature of the structural changes. For HIV-1 Gag assembly, SReD achieved highly sensitive detection without relying on structural priors, significantly outperforming direct analysis of input images. This capability is particularly valuable in studying dynamic cellular processes like viral assembly, where the ability to accurately detect and characterise structures amidst variable backgrounds is crucial. In live-cell imaging of microtubule dynamics, SReD’s multidimensional capabilities allowed for quantitative assessment of structural stability across space and time. This analysis provided novel insights into the differential dynamics of EB3 comets and the microtubule organising centre, demonstrating SReD’s potential for studying complex, time-dependent cellular processes. A key advantage of SReD is its ability to detect and characterise structures without the need for extensive labelled training data or single-molecule localisation input. This feature is particularly useful for exploratory analysis of complex biological systems where the underlying structural patterns may not be fully known *a priori*. The framework’s flexibility in accommodating different reference blocks, from simulated idealised structures to empirically extracted image patches, enhances its utility across diverse experimental scenarios. Another crucial feature is SReD’s robustness to noise and pattern deformations, as demonstrated in our sensitivity analyses. This resilience enables reliable structure detection and quantification even in challenging imaging conditions, expanding the range of biological questions that can be addressed through quantitative image analysis. The algorithm’s multiscale mapping capabilities provide a unique perspective on hierarchical structural organisation, as exemplified by our analysis of nuclear pore complexes at different spatial scales. While SReD offers significant advantages, it is important to acknowledge its limitations. The algorithm’s performance can be influenced by the choice of reference blocks, possibly requiring their optimisation. Additionally, while SReD reduces the need for manual region selection, some level of results curation may still be necessary, particularly in highly complex or heterogeneous samples. Finally, the algorithm’s computational complexity warrants attention. Consider a 2D image with dimensions $n_{1}\times n_{2}$ pixels and a block of size $k_{1}\times k_{2}$ pixels. Each pairwise comparison between the block and an image region requires $O\left(k_{1}k_{2}\right)$ operations. The total number of such overlapping image regions is $\left(n_{1}-k_{1}+1\right)\left(n_{2}-k_{2}+1\right)$. Consequently, the “1-to-all” scheme (block repetition) entails a computational complexity of $O\left(\left(n_{1}-k_{1}+1\right)\left(n_{2}-k_{2}+1\right)k_{1}k_{2}\right)$. When the image dimensions significantly exceed the block size ${(n}_{1}\gg k_{1}$ and $\left.n_{2}\gg k_{2}\right)$, this simplifies to $O\left(n_{1}n_{2}k_{1}k_{2}\right)$. In the “all-to-all” scheme (global repetition), the computational complexity scales quadratically with the image size and linearly with the block size, resulting in $O\left(n_{1}^{2}n_{2}^{2}k_{1}k_{2}\right)$. SReD mitigates this computational burden by harnessing GPU acceleration and pre-calculating background regions that do not warrant analysis. Future developments of SReD could focus on further automating the reference block selection process, potentially incorporating machine learning approaches to optimise block parameters based on image characteristics. Integration with other computational tools, such as deep learning-based segmentation algorithms, could also enhance SReD’s capabilities for more comprehensive structural analysis pipelines.

## Methods

### Noise variance stabilisation.

The Generalised Anscombe Transform (GAT) is applied to the input image to stabilise noise variance, a crucial step in processing microscopy images. These images typically exhibit a combination of Poisson and Gaussian noise, which can obscure the underlying signal. In fluorescence microscopy, noise variance is signal-dependent, limiting some of the assumptions required for the proper application of correlation metrics. Namely, the assumptions of normality, homoscedasticity (equal variance), and absence of outliers. The GAT employs a nonlinear remapping of pixel values, resulting in an output image with near-Gaussian noise and stabilised variance, whilst preserving local contrast and overall image statistics (9). This variance stabilisation thus improves downstream processing, enabling more accurate analysis of the image’s structural content.

### Relevance mask.

We use a relevance mask to filter out areas lacking significant structural information. The rationale is that structural elements present themselves as regional image textures with non-zero variance. Therefore, areas devoid of structure will exhibit minimal texture. Determining the threshold for “minimal” texture is challenging due to the presence of ubiquitous image noise. Rather than choosing an arbitrary value close to zero, we estimated the average noise variance of the input image using a robust estimator (23). This is obtained by sampling the variance of the input image using non-overlapping blocks of the same size as those used for the repetition analysis, and then calculating the average of percentile 0.03. This sampling approach ensures that the noise variance is estimated at the same scale as the analysed structures. The relevance threshold is established by multiplying the estimated average noise variance by an adjustable constant, with the default set at 0. This produces a binary mask outlining areas with sufficient structural content.

### Sampling scheme and mathematical basis.

Our algorithm leverages a custom sampling scheme in which a reference block is compared with all possible test blocks in the image. The scheme can be “1-to-all” or “all-to-all”, depending on the application. The first requires a user-provided reference block, while the latter provides unbiased structure detection. The comparisons between blocks consist of calculating correlation metrics. The correlation metrics can be rotation-variant (e.g., Pearson’s correlation coefficient) or - invariant (e.g., modified cosine similarity). In both cases, the blocks’ dimensions are predefined by the user to match a specific scale. After defining the blocks’ dimensions and the relevance threshold, the algorithm calculates the noise variance-stabilised input, the relevance mask, and normalises the input image to its range. Then, it calculates the local means and standard deviation maps for the entire universe of blocks that can be extracted from the image within the previously defined constraints. To minimise blocking artefacts, the square blocks are transformed into their inbound elliptical counter-parts. Using these statistics, a repetition map is calculated for each “1-vs-all” comparison, where each pixel is assigned a score (named Structural Repetition Score, or SRS), which reflects the similarity between the local neighbourhood centred at that position and the reference block. Finally, the repetition map is normalised to its range. The SRS is given by:

(1) $$SRS\left(X_{i},Y_{j}\right)=Corr\left(X_{i},Y_{j}\right)\cdot Rel\left(Y_{j}\right)$$

where

(2) $$X_{i}=\left\{x_{1},x_{2},\ldots ,x_{n}\right\}$$

and

(3) $$Y_{j}=\left\{y_{1},y_{2},\ldots ,y_{n}\right\}$$

are the reference and test blocks with size n (in pixels) centred at pixel positions i and j, and

(4) $$Rel\left(Y_{j}\right)=\left\{\begin{array}{l} 0,\;\text{if}\;Var\left(Y_{j}\right)\leq \overset{-}{Var}\\ 1,\;\text{if}\;Var\left(Y_{j}\right)>\overset{-}{Var} \end{array}\right.$$

the binary “relevance” label of the test block, where $\overset{-}{Var}$ is the average noise variance of the input image. To analyse local textures and calculate a single value for each, the reference and test blocks require a defined centre. Therefore, the blocks’ dimensions need to be odd, and as a result, $i=\left[\left(r_{h},H-r_{h}\right]\right.$ and $j=\left[r_{w},W-r_{w}\right]$, where $r_{w}$ and $r_{h}$ are the blocks’ width and height radii, and W and H are the input image’s width and height. In the global repetition mode, SReD enables unbiased structure analysis by using the entire universe of image blocks as a reference. Each reference block generates a repetition map that is averaged, and the average value is plotted at the coordinates corresponding to the centre of the reference block. The average uses an exponential weight function based on the distance between the standard deviations of the blocks in each comparison, which enhances structural details. Therefore, the global repetition scores represent the relative repetition of a local texture across the image. Mathematically, the global SRS is given by:

(5) $$S\left(X_{i},Y_{j}\right)=\frac{\sum_{j}\;\;Corr\left(X_{i},\;Y_{j}\right)\;\cdot \;W\left(X_{i},\;Y_{j}\right)\;\cdot \;Rel\left(Y_{j}\right)}{N\;\cdot \;\sum_{j}\;\;W\left(X_{i},\;Y_{j}\right)}$$

where N is the size of the input image (excluding borders), and

(6) $$W\left(X_{i},Y_{j}\right)=e^{-\frac{{\left|\sigma_{X_{i}}-\sigma_{Y_{j}}\right|}^{2}}{\overset{-}{Var}}}$$

is the exponential weight function, where $\sigma_{X_{i}}$ and $\sigma_{Y_{j}}$ are the standard deviations of the reference and test blocks.

### Multiscale analysis.

The scale at which structures are analysed can be adjusted by modulating the ratio between the input image and the block size. For example, larger blocks contain information about higher-order structures compared to smaller blocks. Due to the iterative nature of the algorithm, increasing the block size adds an exponential amount of data points to each comparison, introducing an unwanted load on the computational resources and drastically slowing the calculations. Therefore, modulation of the ratio between the input image and the block size can instead be achieved by adjusting the input image size (i.e., downscaling). A direct consequence of this method is the loss of lower-order information, which should not be problematic when the goal is to increase the sensitivity to higher-order textures.

### Non-linear mapping.

Non-linear mapping can enhance the contrast between different SRSs within the repetition maps, facilitating visual interpretation and subsequent analysis. In our experience, we have found that applying a power transformation to the SRSs often yields the most effective enhancement. This transformation involves raising each SRS value to a specific exponent. The choice of exponent plays a crucial role in determining the degree of contrast enhancement. In this study, we explored a range of exponents between 10 and 10,000. Typically, we initiate the analysis with an exponent of 10 and iteratively adjust it based on the visual assessment of the resulting repetition map. For datasets with subtle structural repetitions or low signal-to-noise ratios, higher exponents may be necessary to amplify the differences between SRSs and reveal hidden patterns. Conversely, for datasets with prominent structural repetitions, lower exponents may suffice to achieve adequate contrast enhancement without introducing excessive noise amplification. The optimal exponent ultimately depends on the specific characteristics of the data and the desired level of visual clarity. By carefully selecting the exponent, users can tailor the contrast enhancement to their needs, facilitating the identification and interpretation of repetitive patterns in diverse microscopy images.

### Optimisation of block parameters for ring pattern detection.

A collection of 248 testing blocks incorporating various combinations of inter-ring spacing and ring height was generated. This process was automated using a custom ImageJ macro. To create input images, five representative segments from the distal axons within each dataset were randomly selected, comprised of six neurons per treatment. These regions were then rotated to align with the horizontal axis to guarantee consistency across subsequent calculations. Then, SReD was used to generate repetition maps for every test block, and their autocorrelation functions were calculated. The relative amplitude of the autocorrelations’ first harmonic was used to assess how effectively each block captured the underlying periodic pattern. The set of block parameter values that maximised the first harmonics’ relative amplitude was systematically identified. The optimised set of parameter values served as a reliable representation of the periodic pattern within the dataset. The optimisation was performed separately for each dataset analysed in this study.

### Detection of reference and virus-like particles using Global Repetition.

An image volume containing 3D simulated reference particles was generated using a custom Python script. The reference particles were added to the input volume by addition. Global Repetition maps were calculated using a block size of 5×5×5 pixels and a relevance constant of 0. Then, the repetition maps were non-linearly mapped using a power transformation with an exponent of 10000. 3D maxima were calculated using the ImageJ “3D Maxima Finder” plugin, with an XY and Z radius of 5 pixels and a minimum threshold of 0.1. The comparison of coordinates between the 3D maxima calculated and the reference particles was performed using a custom Python script.

### Cell culture.

Jurkat cells were cultured in RPMI 1640 (Gibco) supplemented with 10% fetal bovine serum (FBS), 2 mM L-glutamine and 50 μg/mL gentamycin. HEK293T and RPE1-EB3-GFP cells were cultured in DMEM supplemented with 10% fetal bovine serum (FBS), 2 mM L-glutamine and 50 μg/mL gentamycin. Cell lines were cultured at 37°C and 5% CO2.

### DNA plasmids and cell lines.

The RPE1-EB3-GFP cell line was kindly provided by Dr. Mónica Bettencourt-Dias. A plasmid expressing HIV-1 Gag with an internal EGFP tag was generated using the NEB HiFi Assembly Kit (New England Biolabs). A lentiviral backbone containing a tetracycline-inducible promoter and a gene encoding rtTA was prepared by digesting the pCW57.1 plasmid (Addgene #41393) with 5 μg/mL restriction enzymes BamHI and NheI (New England Biolabs) according to the manufacturer’s instructions for 1 hour at 37°C. The digestion product was separated using 1% agarose gel electrophoresis (AGE) and the $\tilde{7}.6$ kb band was purified using the GFX PCR & Gel Band Purification Kit (Sigma-Aldrich) according to the manufacturer’s instructions. Then, three DNA fragments were generated by polymerase chain-reaction (PCR) using Q5 High-Fidelity DNA Polymerase (New England Biolabs). The first fragment (445 bp), encoding the HIV-1 Matrix protein followed by an HIV-1 protease cleavage site (MA-PCS), was generated using Optigag-mNeonGreen-IN (24) as a template and primers *5’-tcagatcgcctggagaattgggccaccatgggtgcgcga-3’ (Fw)* + *5’-ccatacgcgtctggacaatggggtagttttgactgacc-3’ (Rv)*. The second fragment (751 bp), encoding EGFP, was generated using HIV-(i)GFP ΔEnv (20) as a template and primers *5’-ccattgtccagacgcgtatggtgagcaag-3’ (Fw)* + *5’-tagttttgacttctagacttgtacagctcgtc-3’ (Rv)*. The third fragment (1.2 kb), encoding a PCS and the HIV-1 Capsid, Nucleocapsid and p6 proteins (PCS-CA-NC-p6), was generated using Optigag-mNeonGreen-IN (24) as a template and primers *5’-caagtctagaagtcaaaactaccccattgtc-3’ (Fw)* + *5’-aaaggcgcaaccccaaccccgtcattgtgacgaggggtctgaac-3’ (Rv)*. The three fragments were purified using DNA purification columns and their molecular size was confirmed by AGE. The HiFi Assembly reaction was performed using 50 ng of digested vector and equimolar amounts of the three fragments, and incubated at 50°C for 1 hour. The reaction product was diluted 1:4 in dH20, and 2 μL of the dilution was transformed into chemically competent STABL4 bacteria (Thermo Fisher). The bacteria were plated in LB-agar supplemented with 100 μg/mL ampicillin and incubated overnight at 37°C. Several colonies were picked and inoculated into liquid LB containing ampicillin at 100 μg/mL. The plasmid DNA from these colonies was extracted using the GenElute Plasmid Miniprep Kit (Sigma-Aldrich), and was confirmed by digestion with restriction enzyme XbaI followed by AGE (2.3 kb and 7.5 kb fragments). A positive colony was then sequenced using Sanger sequencing (Genewiz) and primers *5’-cgtcgccgtccagctcgacca-3’*, *5’-ccattgtccagacgcgtatggtgagcaag-3’* and *5’-aaaggcgcaaccccaaccccgtcattgtgacgaggggtctgaac-3’*. This process yielded the lentiviral plasmid TetOn-Optigag(i)EGFP, where a human codon-optimised *gag* gene contains a PCS-flanked EGFP-encoding gene. Lentivirus packaging TetOn-Optigag-(i)EGFP were produced to transduce Jurkat cells. To do this, HEK293T cells were cultured in 6-well plates until ~80% of confluence, transfected using 300 μL/well of transfection mixture (DMEM, 3 μg of TetOn-Optigag-(i)EGFP, 1.5 μg of psPAX2 (Addgene #12260), 1.5 μg of CMV-VSV.G (NIAID) and 12 μL of linear polyethyleneimine MW-25,000 (final concentration of 5 μg/μL)(Sigma-Aldrich)) and incubated overnight for 8 hours. Then, the culture medium was replaced with complete DMEM, followed by a 24-hour incubation. The virus-rich supernatant was collected and filtered with 0.22 μm syringe filters. Jurkat cells (2 mL at 1×10^6^ cells/mL) were inoculated with 300 μL of virus-rich supernatant and Polybrene (10 μg/mL), followed by a 3-day incubation. Antibiotic selection of transduced cells was performed by replacing the culture medium with complete RPMI containing puromycin at 2 μg/mL and incubating for 3 days, at which point an “empty virus” control sample had no live cells remaining. The cells were incubated with doxycycline at 1 μg/mL for 24 hours to induce expression and single cells were isolated using Fluorescence-assisted Cell Sorting (FACS). The EGFP-positive population was divided into three subsets according to their relative signal intensity (“Low”, “Medium” and “High”) and single cells were plated into 96-well plates. The cultures were expanded for 15 days and the resulting cell lines were validated using fluorescence microscopy and Western blotting. A clonal line of the “Medium” subset was used for this study.

### Sample preparation and acquisition of microscopy data.

#### HILO imaging of HIV-1 virus-like particle assembly in activated Jurkat cells.

Activation surfaces were prepared based on the protocol in (25). To do this, Lab-Tek 8-well chambers (Thermo Fisher) were cleaned with 100% isopropanol for 10 min and followed by three washing steps with dH_2_0. Then, 200 μL of a hamster anti-CD3 antibody (clone 145.2c11, Creative Biolabs) diluted in PBS at a final concentration of 1 μg/mL was added to the wells and incubated overnight at 4°C. The wells were carefully washed twice with PBS to remove unbound antibodies. Jurkat cells expressing TetOn-Optigag-(i)EGFP were incubated with 1 μM of doxycycline (Sigma-Aldrich) for 24 hours. Then, 50000 cells were added to each well and allowed to adhere and stabilise for 1 hour. Imaging was done in a Nanoimager (ONI) using the 488 nm laser at 10% and channel 0 (two-band dichroic: 498–551 nm and 576–620 nm). The HILO angle was optimised manually and images were acquired at 100 ms exposure. The anti-CD3 antibody was produced at the Flow Cytometry & Antibodies Unit of Instituto Gulbenkian de Ciência, Oeiras, Portugal.

#### 3D imaging of HIV-1 virus-like particle assembly in activated Jurkat cells.

Jurkat cells expressing TetOn-Optigag-(i)EGFP were centrifuged at 200 ×g for 5 minutes and resuspended in complete RPMI containing 0.5 μM of doxycycline to induce Gag expression. Glass coverslips (1.5 mm thick, round, 18 mm diameter) were washed with isopropanol for 10 minutes followed by three washes with dH_2_0. The coverslips were coated with Poly-L-Lysine (PLL, Sigma Aldrich) at 0.1% and incubated for 15 minutes at room temperature, followed by three washing steps with dH_2_0. The PLL-coated coverslips were dried, mounted in an Attofluor chamber (Thermo Fisher) and fixed on the microscope’s stage. The microscope’s enclosure (Okolabs) was heated at 37°C and a manual gas mixer (Okolabs) was used to supply 5% CO_2_. The cells were seeded in the pre-treated coverslips and allowed to settle in the microscope enclosure for 30 minutes. Imaging was performed on an inverted microscope ECLIPSE Ti2-E (Nikon Instruments) equipped with a Fusion BT (Hamamatsu Photonics K.K., C14440–20UP) and a Plan Apo *λ* 100x (NA 1.45) Oil objective. The sample was illuminated with LED light at 515 nm (CoolLED pe800) and acquisition was done at 75 ms exposure with an active Nikon Perfect Focus system and the NIS-Elements AR 5.30.05 software (Nikon Instruments). Volumes were captured by acquiring frames at different depths (z-step size: 0.5 μm). Image deconvolution was performed using a custom Python script based on the Richardson-Lucy method (26, 27) as described in (28, 29).

#### Imaging of EB3-GFP comets in RPE1 cells.

RPE1-EB3-GFP cells (50000 per well) were seeded into Lab-Tek 8-well glass chambers (Thermo Fisher) and allowed to adhere for 24 hours. Imaging was performed in a Nanoimager (ONI) using the 488 nm laser at 10% and channel 0 (two-band dichroic: 498–551 nm and 576–620 nm). Images were acquired at 75 ms exposure for 2 minutes.

#### Assessment of microtubule dynamics using SReD.

Subsets of the original time lapse were created by keeping images belonging to the time frames of interest. Global repetition maps were generated from the temporal subsets using an XY block size of 7×7 pixels, a Z block size equal to the number of images in each subset, and a relevance constant of 0. The repetition maps were non-linearly mapped using a power transformation with an exponent of 1000. NRMSE maps were calculated using the “scikit-image” library (v0.22.0).

## Data Availability

The data obtained in this study is available at https://doi.org/10.5281/zenodo.13764726 under CC BY 4.0 license. The STORM data containing cells with labelled microtubules is available at (10). The data containing DAPI-stained nuclei is available at (11). The STORM data containing nuclear pores with labelled gp210 is available at (12).

## References

[1] Kim Nam Hyeong, Choi Hojae, Shahzad Zafar Muhammad, Ki Heesoo, Lee Jaekyoung, Chae Heeyeop, and Kim Yong Ho. Supramolecular assembly of protein building blocks: from folding to function. Nano Convergence, 9(1):4, January 2022. ISSN 2196–5404. doi: 10.1186/s40580-021-00294-3. 35024976 PMC8755899

[2] Mendes Afonso, Heil Hannah S., Coelho Simao, Leterrier Christophe, and Henriques Ricardo. Mapping molecular complexes with super-resolution microscopy and single-particle analysis. Open Biology, 12(7):220079, July 2022. doi: 10.1098/rsob.220079. Publisher: Royal Society. 35892200 PMC9326279

[3] Aswath Anusha, Alsahaf Ahmad, Giepmans Ben N. G., and Azzopardi George. Segmentation in large-scale cellular electron microscopy with deep learning: A literature survey. Medical Image Analysis, 89:102920, October 2023. ISSN 1361–8415. doi: 10.1016/j.media.2023.102920. 37572414

[4] Ronneberger Olaf, Fischer Philipp, and Brox Thomas. U-Net: Convolutional Networks for Biomedical Image Segmentation, May 2015. arXiv:1505.04597 [cs].

[5] Falk Thorsten, Mai Dominic, Bensch Robert, Çiçek Özgün, Abdulkadir Ahmed, Marrakchi Yassine, Böhm Anton, Deubner Jan, Jäckel Zoe, Seiwald Katharina, Dovzhenko Alexander, Tietz Olaf, Bosco Cristina Dal, Walsh Sean, Saltukoglu Deniz, Tay Tuan Leng, Prinz Marco, Palme Klaus, Simons Matias, Diester Ilka, Brox Thomas, and Ronneberger Olaf. U-Net: deep learning for cell counting, detection, and morphometry. Nature Methods, 16(1):67–70, January 2019. ISSN 1548–7105. doi: 10.1038/s41592-018-0261-2. Publisher: Nature Publishing Group. 30559429

[6] Laine Romain F., Arganda-Carreras Ignacio, Henriques Ricardo, and Jacquemet Guillaume. Avoiding a replication crisis in deep-learning-based bioimage analysis. Nature Methods, 18(10):1136–1144, October 2021. ISSN 1548–7105. doi: 10.1038/s41592-021-01284-3. Publisher: Nature Publishing Group. 34608322 PMC7611896

[7] Heydarian Hamidreza, Schueder Florian, Strauss Maximilian T., Ben van Werkhoven Mohamadreza Fazel, Lidke Keith A., Jungmann Ralf, Stallinga Sjoerd, and Rieger Bernd. Template-free 2D particle fusion in localization microscopy. Nature Methods, 15(10):781–784, October 2018. ISSN 1548–7105. doi: 10.1038/s41592-018-0136-6. Publisher: Nature Publishing Group. 30224671

[8] Heydarian Hamidreza, Joosten Maarten, Przybylski Adrian, Schueder Florian, Jungmann Ralf, van Werkhoven Ben, Keller-Findeisen Jan, Ries Jonas, Stallinga Sjoerd, Bates Mark, and Rieger Bernd. 3D particle averaging and detection of macromolecular symmetry in localization microscopy. Nature Communications, 12(1):2847, May 2021. ISSN 2041–1723. doi: 10.1038/s41467-021-22006-5. Publisher: Nature Publishing Group. PMC8121824 33990554

[9] Boulanger Jérôme, Kervrann Charles, Bouthemy Patrick, Elbau Peter, Sibarita Jean-Baptiste, and Salamero Jean. Patch-Based Nonlocal Functional for Denoising Fluorescence Microscopy Image Sequences. IEEE Transactions on Medical Imaging, 29(2):442–454, February 2010. ISSN 1558–254X. doi: 10.1109/TMI.2009.2033991. Conference Name: IEEE Transactions on Medical Imaging. 19900849

[10] Jimenez Angélique, Friedl Karoline, and Leterrier Christophe. About samples, giving examples: Optimized Single Molecule Localization Microscopy. Methods, 174:100–114, March 2020. ISSN 1046–2023. doi: 10.1016/j.ymeth.2019.05.008. 31078795

[11] Burri Olivier and Guiet Romain. DAPI and Phase Contrast Images Dataset, May 2019.

[12] Diekmann Robin, Kahnwald Maurice, Schoenit Andreas, Deschamps Joran, Matti Ulf, and Ries Jonas. Optimizing imaging speed and excitation intensity for single-molecule localization microscopy. Nature Methods, 17(9):909–912, September 2020. ISSN 1548–7105. doi: 10.1038/s41592-020-0918-5. Publisher: Nature Publishing Group. 32807954 PMC7610360

[13] Leterrier Christophe, Dubey Pankaj, and Roy Subhojit. The nano-architecture of the axonal cytoskeleton. Nature Reviews Neuroscience, 18(12):713–726, December 2017. ISSN 1471–0048. doi: 10.1038/nrn.2017.129. Publisher: Nature Publishing Group. 29097785

[14] Leterrier Christophe. Putting the axonal periodic scaffold in order. Current Opinion in Neurobiology, 69:33–40, August 2021. ISSN 0959–4388. doi: 10.1016/j.conb.2020.12.015. 33450534

[15] Xu Ke, Zhong Guisheng, and Zhuang Xiaowei. Actin, Spectrin, and Associated Proteins Form a Periodic Cytoskeletal Structure in Axons. Science, 339(6118):452–456, January 2013. doi: 10.1126/science.1232251. Publisher: American Association for the Advancement of Science. 23239625 PMC3815867

[16] Vassilopoulos Stéphane, Gibaud Solène, Jimenez Angélique, Caillol Ghislaine, and Leterrier Christophe. Ultrastructure of the axonal periodic scaffold reveals a braid-like organization of actin rings. Nature Communications, 10(1):5803, December 2019. ISSN 2041–1723. doi: 10.1038/s41467-019-13835-6. Publisher: Nature Publishing Group. PMC6925202 31862971

[17] Barabas Federico M., Masullo Luciano A., Bordenave Martín D., Giusti Sebastián A., Unsain Nicolás, Refojo Damián, Cáceres Alfredo, and Stefani Fernando D.. Automated quantification of protein periodic nanostructures in fluorescence nanoscopy images: abundance and regularity of neuronal spectrin membrane-associated skeleton. Scientific Reports, 7(1): 16029, November 2017. ISSN 2045–2322. doi: 10.1038/s41598-017-16280-x. Publisher: Nature Publishing Group. 29167561 PMC5700202

[18] Lavoie-Cardinal Flavie, Bilodeau Anthony, Lemieux Mado, Gardner Marc-André, Wiesner Theresa, Laramée Gabrielle, Gagné Christian, and De Koninck Paul. Neuronal activity remodels the F-actin based submembrane lattice in dendrites but not axons of hippocampal neurons. Scientific Reports, 10(1):11960, July 2020. ISSN 2045–2322. doi: 10.1038/s41598-020-68180-2. Publisher: Nature Publishing Group. 32686703 PMC7371643

[19] Masenga Sepiso K., Mweene Bislom C., Luwaya Emmanuel, Muchaili Lweendo, Chona Makondo, and Kirabo Annet. HIV–Host Cell Interactions. Cells, 12(10):1351, January 2023. ISSN 2073–4409. doi: 10.3390/cells12101351. Number: 10 Publisher: Multidisciplinary Digital Publishing Institute. 37408185 PMC10216808

[20] Hübner Wolfgang, Chen Ping, Del Portillo Armando, Liu Yuxin, Gordon Ronald E., and Chen Benjamin K.. Sequence of Human Immunodeficiency Virus Type 1 (HIV-1) Gag Localization and Oligomerization Monitored with Live Confocal Imaging of a Replication-Competent, Fluorescently Tagged HIV-1. Journal of Virology, 81(22):12596–12607, November 2007. ISSN 0022–538X. doi: 10.1128/JVI.01088-07. 17728233 PMC2168995

[21] Floderer Charlotte, Masson Jean-Baptiste, Boilley Elise, Georgeault Sonia, Merida Peggy, El Beheiry Mohamed, Dahan Maxime, Roingeard Philippe, Sibarita Jean-Baptiste, Favard Cyril, and Muriaux Delphine. Single molecule localisation microscopy reveals how HIV-1 Gag proteins sense membrane virus assembly sites in living host CD4 T cells. Scientific Reports, 8:16283, November 2018. ISSN 2045–2322. doi: 10.1038/s41598-018-34536-y. 30389967 PMC6214999

[22] Stepanova Tatiana, Slemmer Jenny, Hoogenraad Casper C., Lansbergen Gideon, Dortland Bjorn, De Zeeuw Chris I., Grosveld Frank, van Cappellen Gert, Akhmanova Anna, and Galjart Niels. Visualization of Microtubule Growth in Cultured Neurons via the Use of EB3-GFP (End-Binding Protein 3-Green Fluorescent Protein). Journal of Neuroscience, 23(7):2655–2664, April 2003. ISSN 0270–6474, 1529–2401. doi: 10.1523/JNEUROSCI.23-07-02655.2003. Publisher: Society for Neuroscience Section: ARTICLE. 12684451 PMC6742099

[23] Liu Wenjiang, Liu Tao, Rong Mengtian, Wang Ruolin, and Zhang Hao. A fast noise variance estimation algorithm. In 2011 Asia Pacific Conference on Postgraduate Research in Microelectronics & Electronics, pages 61–64, October 2011. doi: 10.1109/PrimeAsia.2011.6075071. ISSN: 2159–2160.

[24] Mamede João I., Griffin Joseph, Gambut Stéphanie, and Hope Thomas J.. A New Generation of Functional Tagged Proteins for HIV Fluorescence Imaging. Viruses, 13(3):386, March 2021. ISSN 1999–4915. doi: 10.3390/v13030386. Number: 3 Publisher: Multidisciplinary Digital Publishing Institute. 33670986 PMC7997544

[25] Ashdown George, Pandžic Elvis, Cope Andrew, Wiseman Paul, and Owen Dylan. Corti-´ cal Actin Flow in T Cells Quantified by Spatio-temporal Image Correlation Spectroscopy of Structured Illumination Microscopy Data. Journal of Visualized Experiments (JoVE), (123): e55617, December 2015. doi: 10.3791/53749. PMC4694038 26709554

[26] Richardson William Hadley. Bayesian-Based Iterative Method of Image Restoration*. JOSA, 62(1):55–59, January 1972. doi: 10.1364/JOSA.62.000055. Publisher: Optica Publishing Group.

[27] Lucy L. B.. An iterative technique for the rectification of observed distributions. The Astronomical Journal, 79:745, June 1974. ISSN 0004–6256. doi: 10.1086/111605. Publisher: IOP ADS Bibcode: 1974AJ.....79..745L.

[28] Carter Stephen D., Mamede João I., Hope Thomas J., and Jensen Grant J.. Correlated cryogenic fluorescence microscopy and electron cryo-tomography shows that exogenous TRIM5α can form hexagonal lattices or autophagy aggregates in vivo. Proceedings of the National Academy of Sciences, 117(47):29702–29711, November 2020. doi: 10.1073/pnas.1920323117. Publisher: Proceedings of the National Academy of Sciences. PMC7703684 33154161

[29] Yoh Sunnie M., Mamede João I., Lau Derrick, Ahn Narae, Sánchez-Aparicio Maria T., Temple Joshua, Tuckwell Andrew, Fuchs Nina V., Cianci Gianguido C., Riva Laura, Curry Heather, Yin Xin, Gambut Stéphanie, Simons Lacy M., Hultquist Judd F., König Renate, Xiong Yong, García-Sastre Adolfo, Böcking Till, Hope Thomas J., and Chanda Sumit K.. Recognition of HIV-1 capsid by PQBP1 licenses an innate immune sensing of nascent HIV-1 DNA. Molecular Cell, 82(15):2871–2884.e6, August 2022. ISSN 10972765. doi: 10.1016/j.molcel.2022.06.010. 35809572 PMC9552964

[30] Otsu Nobuyuki. A Threshold Selection Method from Gray-Level Histograms. IEEE Transactions on Systems, Man, and Cybernetics, 9(1):62–66, January 1979. ISSN 2168–2909. doi: 10.1109/TSMC.1979.4310076. Conference Name: IEEE Transactions on Systems, Man, and Cybernetics.

[31] Meijering E., Jacob M., Sarria J.-C.f., Steiner P., Hirling H., and Unser M.. Design and validation of a tool for neurite tracing and analysis in fluorescence microscopy images. Cytometry Part A, 58A(2):167–176, 2004. ISSN 1552–4930. doi: 10.1002/cyto.a.20022. _eprint: 10.1002/cyto.a.20022. 15057970

